# Who Believes in ESP: Cognitive and Motivational Determinants of the Belief in Extra-Sensory Perception

**DOI:** 10.5964/ejop.v15i1.1689

**Published:** 2019-02-28

**Authors:** Marija Branković

**Affiliations:** a Department of Psychology, Faculty of Media and Communications, Singidunum University, Belgrade, Serbia; University of Belgrade, Belgrade, Serbia

**Keywords:** paranormal belief, extra-sensory perception, intuition, fear of death, fatalism

## Abstract

Many people believe in extra-sensory perception, e.g. the ability to communicate with thoughts, to sense future events or locate radiation with the help of a V-shaped piece of wood. Addressing a gap in research specifically focused on ESP beliefs, we investigated cognitive styles and basic motivations related to these beliefs in two survey studies. The findings suggest that a propensity to use intuition is the best predictor of ESP beliefs in terms of cognitive style. ESP belief is positively related to fear of death, and this relation is partly mediated by fatalism, i.e. the belief that chance controls one’s life. ESP beliefs do not seem to be perceived as irreconcilable with a rational view of reality however, they do not necessarily provide psychological protection from existential concerns. The implications of the findings in terms of costs and benefits of these beliefs and the possibility to change them are discussed.

The *One Million Dollar Paranormal Challenge* was an offer of 1 million US dollars to anyone who can demonstrate a paranormal ability under scientific testing conditions^i^. From 1964 to 2015, when the competition was terminated, not a single person of over thousand applicants succeeded in proving their supernatural ability. However, recent studies reveal that around two-thirds of Americans believe in psi phenomena (Rice, 2003). ESP or “psi” refers to extra-sensory perception, i.e. phenomena as telepathy (communicating with thoughts), psychokinesis (the ability to move objects without physical contact), precognition (the ability to predict future events), psychometry (reading the past from an object) or dowsing (the ability to locate underground water, buried metals and gravesites using dowsing rod). The common denominator for all the phenomena in question is that they break the fundamental scientific principles known to date (Broad, 1953). The most recent attempts to demonstrate the reality of ESP were made by Daryl Bem (Bem, 2011), but several labs promptly attempted and failed to replicate his findings (Galak, LeBoeuf, Nelson, & Simmons, 2012; Ritchie, Wiseman, & French, 2012). Since they failed to stand numerous scientific tests (e.g. Enright, 1995) these phenomena are thus considered pseudoscientific or, as other authors prefer to term this “…not empirically attested to the satisfaction of the scientific establishment.” (Irwin, 2009, p. 16).

The high prevalence of ESP beliefs, even among well-educated individuals (Rice, 2003; Wuthnow, 1978), calls for a better understanding of their psychological determinants. In two studies we, therefore, investigated cognitive and motivational determinants of ESP beliefs, more precisely which kind of cognitive style predicts these beliefs and whether they are deeply founded in some basic existential concerns, i.e. fear of death.

## ESP as a Type of Paranormal Belief

Paranormal belief is a term with very wide and varying content. The most widely used instrument for studying paranormal beliefs (Tobacyk & Milford, 1983, also a revised version Tobacyk, 2004) has been extensively criticized for grouping together different types of beliefs that have different origins and different correlates (Aarnio & Lindeman, 2005; Lawrence, 1995; Rice, 2003; Thalbourne, 1995; Wiseman & Watt, 2004), e.g., belief in God and other religious beliefs, beliefs in ghosts, supernatural healing, precognition, superstition etc. Although authors in the field appear to agree that paranormal belief is a multidimensional phenomenon (Aarnio & Lindeman, 2005; Irwin, 1993, 2009) the exact number and nature of the relevant dimensions are yet to be established. However, in previous studies, factor analytical analyses have often identified a component that is related to psi or similar phenomena (e.g. Lange, Irwin, & Houran, 2000; Tobacyk & Milford, 1983).

Beliefs in ESP phenomena are interesting because they are seemingly a more “modern” form of paranormal belief, perhaps more in line with the current worldviews, compared to more traditional forms of superstitious beliefs or religious beliefs. For instance, Schouten (1983) found that students espousing ESP beliefs did not express negative feelings about the influence of technology in the modern society (see also Wuthnow, 1978). Supporting this, a recent survey conducted on a representative sample of the US public revealed that 60% of participants expressed their belief in ESP, which makes them one of the most prevalent forms of paranormal belief (in comparison with 33% who believe in astrology, 35% believing that extraterrestrials visited the Earth in the past, or 24% acknowledging that they are at least somewhat superstitious; Rice, 2003; see also Irwin, 2009). Another finding illustrating the prevalence of ESP beliefs is that people tend to interpret their unusual experiences in life in terms of psi, although in most cases it is possible to rule out this interpretation (Kennedy, 2005).

Traditionally, researchers interested in ESP came mostly from the ranks of parapsychologists and their interest was primarily related to the issue of how belief in ESP affects performance in ESP tasks (Irwin, 2009). They studied the *sheep-goat effect*, that is, the phenomenon that persons who believe in ESP (sheep) are also more successful at tasks created to demonstrate ESP phenomena, compared to skeptics (goats) (e.g. Storm & Thalbourne, 2005; Thalbourne, 2010). Otherwise, most of the previous research was related to paranormal beliefs in general, and thus have a limited applicability to ESP beliefs specifically.

In this study, we therefore decided to focus on this specific type of paranormal belief and study some of its psychological foundations. A more thorough understanding of ESP beliefs has implications for the wider debate related to personal, social and political consequences of holding and acting upon such beliefs (see also Irwin, 2009). It would also help better delineate the relations of these specific beliefs and the more general category of paranormal belief.

## The Present Study

In this manuscript, we will present two studies. Study 1 examined whether ESP beliefs can be reliably measured and whether rational or intuitive cognitive styles are better predictors of these beliefs. Study 2 examined the motivational foundations of ESP beliefs, in particular, their relation to fear of death and external/internal locus of control. As already argued, since research on ESP has been largely integrated within the study of paranormal belief in general, we will start by presenting the findings from this wider framework and then discuss whether they also apply to ESP, theoretically and (if possible) empirically.

## Study 1

### Theoretical Rationale

#### Cognitive Predictors of Paranormal Belief

Previous research suggests that socio-demographic differences account for a very small percentage of the variance in paranormal belief, disproving the “deprivation theory”, i.e. the idea that poorer educational and socio-cultural background should make individuals more susceptible to paranormal belief (Rice, 2003, cf. Haraldsson, 1981; Pennycook, Cheyne, Seli, Koehler, & Fugelsang, 2012; Wuthnow, 1978). The existent research also failed to reveal deficits in critical reasoning ability among paranormal believers (Hergovich & Arendasy, 2005; Roe, 1999). Portions of research suggest that it is the individual’s cognitive style, rather than cognitive ability or education that makes the difference. A propensity for an analytical thinking style has been shown to negatively predict paranormal belief although this link has been studied most extensively with regard to religiousness or using the undifferentiated measures of paranormal belief (Gervais & Norenzayan, 2012; Morgan, 2016; Pennycook et al., 2012; Pennycook, Ross, Koehler, & Fugelsang, 2016). For instance, Pennycook and colleagues recently presented a meta-analytical integration of a number of studies which go to show that non-believers are more analytical and reflexive than believers (Pennycook et al., 2016). The authors argue that a propensity for analytical thinking undermines religiosity and other kinds of paranormal belief because people prone to analytical thinking are readier to critically examine culturally accepted beliefs and renounce them (Pennycook et al., 2012, 2016).

Paranormal belief has also been related to more specific deficiencies in rational thinking, such as misperception of chance (Blackmore & Troscianko, 1985; Brugger, Landis, & Regard, 1990; Dagnall, Parker, & Munley, 2007) or confusion of ontological domains (Lindeman & Aarnio, 2007). However, the existent research suggests that a lack of rationality cannot be the exclusive explanation since a large percentage of variance still remains unexplained (Pennycook et al., 2016; cf. Gray, 1985). Furthermore, different authors, especially developmental psychologists, argue for the important role of intuitive thinking in development and maintenance of paranormal belief, religious belief in particular (Boyer, 2008; Epley, Converse, Delbosc, Monteleone, & Cacioppo, 2009; Kelemen, 2004). In line with this, intuitive cognitive style has been demonstrated to predict esoteric thinking and superstition (Epstein, Pacini, Denes-Raj, & Heier, 1996). Lindeman and Aarnio (2007) also found that intuitive thinking was the more important predictor of superstition and paranormal belief than analytical thinking.

#### Cognitive Styles and ESP Belief

A central issue of interest for the present study is whether the previous findings can be extrapolated to ESP beliefs in particular. Similar to other paranormal belief, belief in ESP was found not to be related to the reasoning ability (Hergovich & Arendasy, 2005), suggesting that it is differences in cognitive style rather than cognitive ability that are of importance. Furthermore, it has been established that better-educated individuals are in fact *more* likely to endorse belief in psi (Rice, 2003). It is possible that ESP beliefs have some specific relations to cognitive style. First, it is possible that an analytical cognitive style can be related to an interest in and espousing of ESP beliefs, as an alternative to more traditional religious and superstitious ones (cf. Morgan, 2016). Another issue of interest is the relationship between analytic and intuitive cognitive styles. In the cited research analytical style has most frequently been defined as the propensity to overcome highly salient intuitive solutions to problems (Pennycook et al., 2012), and measured with the cognitive reflection task (Frederick, 2005). This conceptualization has two tacit assumptions, namely that the two cognitive styles exclude each other and that the preferred styles identified in a problem solving context can be generalized to other issues and domains. However, it is possible that a person with a highly analytical cognitive style in rational problem solving would still lean on the intuition when thinking about whether there is more to reality than we can perceive.

We, therefore, chose to investigate both intuitive and rational cognitive styles. Based on dual system models of information processing (Evans, 2003; Kahneman, 2015; Petty & Cacioppo, 1986), Pacini and Epstein (Epstein et al., 1996; Pacini & Epstein, 1999) distinguish between a rational (analytical, objective, fact-oriented) mode and an experiential (intuitive, associative, emotional) mode of information processing. The core proposition of the model is that the two systems operate *independently* so that individuals can hold conflicting beliefs arising from the two different systems (e.g. death is an irreversible ending of life / the soul continues to exist after death). Following this distinction, belief in ESP could be more closely related to the operation of the intuitive than the (inconsistency) of the rational system, resulting in ESP beliefs existing side-by-side with rational and scientifically based worldviews. In the current study, we wanted to investigate whether analytical or experiential style are significant predictors of ESP beliefs as well as which style contributes more to their prediction.

### Method

#### Participants and Procedure

Two hundred and fifty-seven students from the Faculty of Philosophy, Faculty of Media and Communications and Faculty of Mathematics^ii^ in Belgrade participated in a survey study (43% female, mean age 21.94, *SD* = 5.74). Roughly a half of the students (58%) were administered a pen-and-paper questionnaire during classes at the university, while the remaining participants responded online. Students participated voluntarily and signed (clicked on) informed consent prior to answering the questionnaire.

#### Instruments

The questionnaire consisted of a short socio-demographic section and three scales that were counterbalanced, to prevent any order effects.

##### Extra-sensory perception belief scale

was developed, which consisted of 12 items with 5-point rating scales. We chose to examine belief in most common phenomena related to ESP: telepathy, precognition, dowsing and perception of causality instead of chance. The scale tapped into phenomena close to the everyday experience that could be interpreted as evidencing ESP (e.g. *I believe that it is not a coincidence that when I intend to call someone, that very person calls me*. Or *I always feel when a close person is not feeling well, even when we do not have direct contact*). The scale showed good internal consistency (α = .85).

Principal component analysis revealed a clear unidimensional structure of this scale (detailed in Table 1). The first principal component explained 38.7% of the variance and had high loadings (a minimum of .40) from all the items in the scale. The second component explained additional 9.82% of the variance, and it appears to be a more specific aspect of ESP belief, most closely related to “sensing” events or people without direct contact. Inspection of the Scree plot suggests that the largest difference in the percentage of the explained variance is between the first and second component (the third component explained 8.03% of variance), so we therefore conclude that the one-factor solution is the most adequate for this scale.

**Table 1:** Factor Loadings for the First Principal Component Extracted From the Scale of ESP Beliefs

Item	Factor loading
I believe that some people can sense future events.	.747
I believe that is not a coincidence when the very person I am thinking about calls me.	.741
The alleged parapsychological powers boil down to pure speculation or fraud.	.717
With the help of certain instruments (as dowsing rod), people can detect sources of dangerous radiation in the house.	.652
I think that the modern science has shown there is no evidence for parapsychological claims.	.649
I believe it is possible for people to sense things from the domains beyond their physical senses.	.625
When I guess correctly the side on which a coin will lend, I know that it is a result of pure chance.	.615
I can sense when somebody is watching me from behind.	.610
I believe that profilers have the ability to read the circumstances of a crime from objects.	.562
Even though I do not know how, I can always feel when a close person is unwell, without any direct contact.	.537
Sometimes I dream about things that later happen.	.523
I do not believe that dowsing (using metal or tree V- shaped instruments) is a reliable method of detecting underground water.	.409

##### Superstition

was measured by the scale developed in Serbian and validated by Žeželj, Pavlović, Vladisavljević, and Radivojević (2009). This scale taps into the most frequent traditional forms of superstitious beliefs, e.g. *When somebody mentions some unfortunate event, it is good to knock on wood, for protection.* Or *I never go under a ladder, even when this is more convenient for me.* It has 20 items with 5-point rating scales (α = .89).

##### Cognitive styles

were measured by a translated version of the 40-item *Rational-Experiential Inventory* (REI; Pacini & Epstein, 1999). This inventory assesses rational and experiential styles through two dimensions: engagement (motivation to use rational and intuitive thinking) and self-rated ability. Rational style is indicated by the endorsement of items as *I am much better at figuring things out logically than most people (ability)* or *Using logic usually works well for me in figuring out problems in my life* (engagement). Intuitive style is indicated by items as *When it comes to trusting people, I can usually rely on my gut feelings* (ability) and *Intuition can be a very useful way to solve problems* (engagement).

A principal component analysis was conducted to explore the structure of the scale since we did not find any previous report using the scale in Serbian translation. The first two components (18.11%, 16.73%) could clearly be interpreted as experiential and rational cognitive styles, since they had loadings from all the respective items from the scales. Scree plot suggested that the largest difference in explained variance was between the second and the third factor (which explained 5.49% of the variance), so a two-factor solution appears to be the best fitted to the data. However, since the subscales suggested by the authors provided reliable measures of the described dimensions (α = .75 for RA, .78 for RE, .82 for EA, and .78 for EE), and they offered the distinction relevant to the current research question, we used them in the analyses to allow more precise conclusions^iii^.

### Results

Descriptive statistics and correlations between variables are presented in Table 2.

**Table 2:** Descriptive Statistics and Correlations Between Variables in Study 1

Variable	*M*	*SD*	Correlations					
			1	2	3	4	5	6
1. ESP beliefs	2.90	.75	–	.58**	-.12*	-.05	.32**	.43**
2. Superstition	2.13	.72		–	-.20*	-.31**	.20**	.25**
3. Rational ability	3.81	.58			–	.69**	.14	-.09
4. Rational engagement	3.69	.69				–	.12*	-.05
5. Experiential ability	3.29	.68					–	.69**
6. Experiential engagement	2.97	.68						–

*Note.* We report Spearman coefficients since the variables deviated from normal distribution.

*p < .05. **p < .01.

While there is a substantial correlation between the superstition and ESP belief scale scores, belief in ESP is endorsed significantly more than the traditional forms of superstitious beliefs, *t*(243) = 16.33, *p* < .001.

Both types of beliefs are related to the dimensions of cognitive styles, as evidenced by the presented correlations. However, it is predominantly the dimensions of the experiential rather than the rational cognitive styles that are related to ESP beliefs. We further explored the ability of cognitive styles to predict ESP beliefs by way of a regression analysis.

Regression analyses revealed that cognitive styles explained 20.4% of the variance in ESP beliefs and that the best predictor was intuitiveness, more precisely the motivation to use intuition (β = .35, *p* < .001) (detailed in Table 3). The self-rated rational ability was only marginally significant as a predictor (β = -.15, *p* = .066). In comparison, cognitive styles explained 16% of the variance in superstition, but the predictors that emerged significant were different: traditional superstitious beliefs are best predicted by a lack of rational engagement (β = -.27, *p* = .001) and the self-rated experiential ability (β = .20, *p* = .018).

**Table 3:** Results From Multiple Regression Analyses in Study 1

Criterion / Predictor	B	*SE*	β	*p*
ESP				
Constant	1.75	0.39	–	< .001
Rational ability	-0.19	0.10	-.15	.066
Rational engagement	0.06	0.08	.06	.467
Experiential ability	0.12	0.90	.10	.216
Experiential engagement	0.42	0.10	.35	< .001
Superstition				
Constant	2.49	0.39	–	< .001
Rational ability	-0.08	0.10	-.06	.452
Rational engagement	-0.29	0.08	-.27	.001
Experiential ability	0.22	0.90	.20	.018
Experiential engagement	0.10	0.10	.08	.340

### Discussion

In the present study, we set out to investigate beliefs in extra-sensory perception, as one specific type of the wider category of paranormal beliefs. We argued that, in addition to further study of the multiple dimensions of paranormal belief and their mutual relations (Irwin, 2009), an approach focusing on specific types of these beliefs can be warranted. Our short scale developed to measure ESP beliefs proved a reliable and relatively unidimensional instrument so we believe its further use and refinement can be recommended. To establish convergent and discriminant validity of this scale, we compared it to a reliable measure of more traditional forms of superstitious belief (Žeželj et al., 2009). We have seen that the scales do correlate to a considerable extent but also that the average scores are higher on ESP beliefs than superstition scale, which means that these beliefs appear to our young participants as more acceptable than the traditional ones. Importantly, superstition and ESP beliefs showed distinct patterns of relations with the aspects of cognitive styles: while intuitive engagement predicted ESP, a lack of rational engagement and self-rated intuitive ability predicted superstitious beliefs. We can thus conclude that ESP beliefs are a phenomenon related to other types of paranormal belief (superstition) but can be recognized as a distinct and seemingly more acceptable type, more in line with the modern life. A clear limitation of the present analysis is the fact that the superstition and ESP scales have been developed independently, so that we can observe some overlaps in their contents (e.g. related to foreseeing the future). Future studies need to distinguish more clearly between these two dimensions, to achieve optimal discriminative validity.

Now turning to the main issue of the present study, our findings reveal that a propensity to rely on intuition is a more important predictor of ESP belief than a lack of rationality. This is in line with some of the previous research theoretically founded in the idea of rationality and intuitiveness as independent rather than contrasting thinking styles (Lindeman & Aarnio, 2007). At the same time, our findings complement previous studies that defined a rational thinking style (more precisely, ability) as the propensity to overcome intuitive responses (Pennycook et al., 2012). An obvious difference is that our measures relied on self-report rather than more objective assessment. It can be argued that the fact that rational ability did not emerge as a predictor of ESP depends on the nature of the measure, which does not have to reflect more objective measures of ability. This remains an issue to be addressed by future studies that should focus more specifically on ESP beliefs. Another interesting issue would be examining intuitive ability, however, this appears to be a much more elusive construct, difficult to conceptualize and measure objectively.

Our findings thus lend support to a relative independence of intuitive and rational cognitive styles, both structurally (in terms of principal components) and functionally (as predictors of different types of beliefs). Apparently, ESP belief can exist side by side with rational and scientific worldviews within the same individual. This could be either because some individuals do not acknowledge a sharp dividing line between these worldviews or, quite contrary, that they do recognize this division and keep their intuitive approach for the domains other than science. As an example of the former stance, we can consider Daryl Bem, the scientist we mentioned earlier in this paper. He is at the same time an enthusiastic proponent of parapsychological research and a scientist who adheres to stringent scientific principles of research. An example of the second stance is the approach called NOMA (Non-overlapping magisteria; Gould, 1997) advocating treating science and religion as independent fields – science as providing facts and religion as the domain of morality, values and meaning. Future studies should investigate how and why individuals choose to combine this seemingly opposite views. Perhaps these worldviews fulfill different psychological needs of the individual. In the following study, we will focus on some of these needs and motives.

## Study 2

### Theoretical Rationale

#### The Motivational Underpinnings of Paranormal Belief

The ubiquity of paranormal beliefs opens up the question of whether they could have some important psychological functions, i.e. serve some basic psychological needs. Existential motives have been proposed as basic motivations of paranormal belief, i.e. the need for meaning in life, for overcoming uncertainty, establishing (an illusion of) control or diminishing fear of death (Irwin, 1993, 2009; Kennedy, 2005). A frequent motivational account of paranormal belief is that it is primarily motivated by a desire to achieve control over the unusual or uncertain aspects of life (Irwin, 1993). For instance, Rudski and Edwards (2007) examined the use of superstitious rituals in the everyday life of students and concluded that these rituals mostly occur in uncertain situations, thus providing the students with an illusion of control and a way of coping. Following the same basic idea, an interesting study of the occurrence of psychological papers examining parapsychology through several decades (from 1929 to 1977) found that unfavorable social conditions (expressed through both subjective and objective indicators) predicted scientific interest in parapsychology (McCann & Stewin, 1984). Previous findings also suggest a relation between paranormal belief and the external locus of control, i.e. the tendency to ascribe events in one’s life to external forces (Dag, 1999; Groth-Marnat & Pegden, 1998; Tobacyk & Tobacyk, 1992), although this was not demonstrated for the psi-subscale of the general paranormal belief (e.g. Groth-Marnat & Pegden, 1998).

Previous research also established a link between paranormal belief and death anxiety (Irwin, 1993), however, there is still controversy as to whether this relationship is positive or negative. In an experimental study, it was found that making thoughts about personal mortality salient lead participants to report more belief in supernatural agents, coming from both one’s own and other religions, as well as a strengthened belief in divine intervention (Norenzayan & Hansen, 2006). From the opposite perspective, Kennedy and Kanthamani (1995) found that participants who thought they had paranormal and/or transcendent experiences reported a stronger belief in life after death, belief in a higher power and sense of purpose in life as well as decreased fear of death. The authors, however, did not clearly distinguish what they termed paranormal and transcendent experiences and in particular transcendent experiences appear to be defined circularly, so that the effects of the experience cannot be delineated from the experience itself (see p. 251).

#### Motivations Underlying ESP Beliefs

In this study, we chose to focus on the relation of ESP beliefs with death anxiety and the locus of control, as representative of the most common underlying motivations suggested by previous research. Theoretically, it can be argued that these motivations are also relevant for ESP belief, that is, that ESP is motivated by a desire to better control one’s environment and reduce some basic anxieties. What is more, since death is one of the ultimately non-controllable aspects of life, need for control and fear of death appear to be related. This proposition has received empirical support from other domains of research. In one study (Fritsche, Jonas, & Fankhänel, 2008) the authors showed that death anxiety can be diminished when participants are induced to perceive some level of personal control over the circumstances of the process of dying as opposed to those who did not. These findings suggest that the need for control could be one of the crucial components of death anxiety.

Contrary to more traditional superstitious and religious beliefs, Davies and Kirkby (1985) show that belief in psi is related to an internal locus of control, at least regarding personal and interpersonal spheres. Irwin (2000) also found that a component he interpreted as psi beliefs may be related to a heightened desire for control and a conviction that one has the means to control the events in the sociopolitical arena. The latter finding is difficult to interpret, so the author suggests its further validation. It is apparent from this short review that the relation between the locus of control and ESP belief needs further investigation with more focused and reliable measures.

However, it is questionable whether belief in psi can offer psychological certainty one strives for. Some studies suggest that potentially protective function of paranormal belief in facing existential uncertainties and fears could be limited to religious beliefs. In line with this, Tobacyk and Pirttilä-Backman (1992) found a *positive* correlation between death anxiety and the dimension of paranormal belief related to psi, at least among their Finnish participants. Wong (2009) proposes a sort of a vicious circle of superstition and anxiety in which anxiety leads to superstition, which cannot relieve the anxiety, thus leading to it becoming even more intensive, which in turn leads to more superstition and so on. This reasoning might also be applicable to ESP belief. Psi phenomena suggest a certain alternative perspective on reality and it has been shown that people tend to explain their unusual experiences in terms of psi (Kennedy, 2005). However, this is not comparable to the sort of more organized worldviews as religion, with clear values and standards of behavior, and coming with a promise of literal immortality. Therefore, these beliefs might not offer sufficient comfort in facing basic anxiety.

Based on these combined insights, we wanted to test the idea that ESP beliefs are related to basic existential anxieties, more precisely to the individual’s chronic level of fear of death. Based on previous research suggesting that control is one of the most important aspects of death anxiety (Fritsche et al., 2008), we also wanted to examine whether the locus of control would mediate this relationship, that is, whether a tendency to attribute outcomes in one’s life to chance would mediate the relationship between fear of death and ESP beliefs.

We expect that fatalism should mediate the effect of fear of death on ESP beliefs, since fear of death is proposed to be the basic existential concern, which shapes the way in which a person perceives the environment (Pyszczynski, Greenberg, & Solomon, 1997). If fear of death gives rise to a need to control the environment and perceive it as less uncertain, fatalism could be one of possible responses or paths through which some people deal with this basic fear (e.g. believing in destiny alleviates at least some of the fear in dealing with the uncertain future). However, it is also possible that people more prone to fatalism experience more fear of death, which, in turn, motivates them to seek to believe in something “more” that is out there (e.g. psi phenomena). Therefore, we will also test this alternative mediation model (from fatalism, through fear of death, to ESP beliefs).

### Method

#### Participants and Procedure

Two hundred and twenty students from the Faculty of Media and Communications, Faculty of Philosophy and Faculty of Mathematics in Belgrade^iv^ were surveyed (66.8% female, mean age 21.34, *SD* = 3.49). The surveys were administered during classes at the university. Participants read and signed informed consent prior to participation. They were thanked and provided with either oral or written debriefing.

#### Instruments

##### ESP beliefs

were assessed using the scale described in the previous study (α = .83). Principal component analysis suggested a single-factor solution, with almost the exact same percentage of explained variance as in Study 1 (38.64%).

##### Fear of death 

was measured by the 28-item Collet-Lester Fear of Death Scale (Lester & Abdel-Khalek, 2003) (α = .92). The scale was translated for the purposes of the present study by two independent translators, while the final formulations were agreed upon through discussion. No substantial change was made in comparison to the original item wording. Participants indicated the degree to which they found different aspects of death and the process of dying troubling, on a 5-point rating scales. According to the authors, the scale consists of four subscales: own death, own dying, death of (close) others and dying of (close) others. A principal component analysis was performed to explore the structure of the scale. The first component explained the largest proportion of variance (32.85%) and had high loadings (> .46) from all the items included in the scale. Also, the largest difference in the percentage of the explained variance was between the first and the second component (second component explained 9.58%, third component 6.59%), suggesting a one-factor solution. We thus computed a total score indicating the level of fear of death.

##### Locus of control

was measured by the multi-dimensional IPC (Internality, Powerful Others, and Chance Scales) (Levenson, 1981), a 24-item instrument with 6-point rating scales, ranging from -3 (*do not agree at all*) to +3 (*fully agree*). This scale was intended to offer a more differentiated measure of locus of control, an individual’s chronic tendency to interpret the events in his or her life as mostly dependent on the individual him- or herself (internal locus) or dependent on external factors, such as chance (fatalism) or the will of powerful others. A principal component analysis suggested that the first extracted component explained the largest percentage of variance (25.59%) while the second and the third component explained 10.40% and 7.40% of variance, respectively. Factor loadings suggest that there is a common, bi-polar dimension, underlying this construct. However, since the previous research highlights the role of the perception of chance or fatalism as a specifically important determinant of paranormal belief, we computed the scores for the three subscales, internality (α = .68)^v^, powerful others (α = .84), and chance/fatalism (α = .73), according to the original instruction (Levenson, 1981) and divided the sums with the number of items.

### Results

Descriptive statistics and correlations between variables are presented in Table 4.

**Table 4:** Descriptive Statistics and Correlations Between Variables in Study 2

Variable	*M*	*SD*	Correlations				
			1	2	3	4	5
1. ESP beliefs	3.09	.76	–	.28**	.05	.29**	-.03
2. Fear of death	3.40	.73		–	.16**	.33**	-.12*
3. IPS powerful others	1.41	.91			–	.43**	-.42**
4. IPS fatalism	2.51	.98				–	-.29**
5. IPS internal	1.93	.63					–

*Note.* We report Spearman coefficients since the variables deviated from normal distribution.

*p < .05. **p < .01.

As can be seen from the table, belief in ESP was positively related to fear of death and the dimension of the external locus of control related to the perception of chance / fatalism, but not to the dimension of *powerful others* or internality. Also, fear of death was positively related to fatalism as well as the other external dimension of “powerful others” while being negatively related to internal locus of control, in line with previous research relating fear of death to a lowered sense of control (Fritsche et al., 2008).

**Figure 1:**
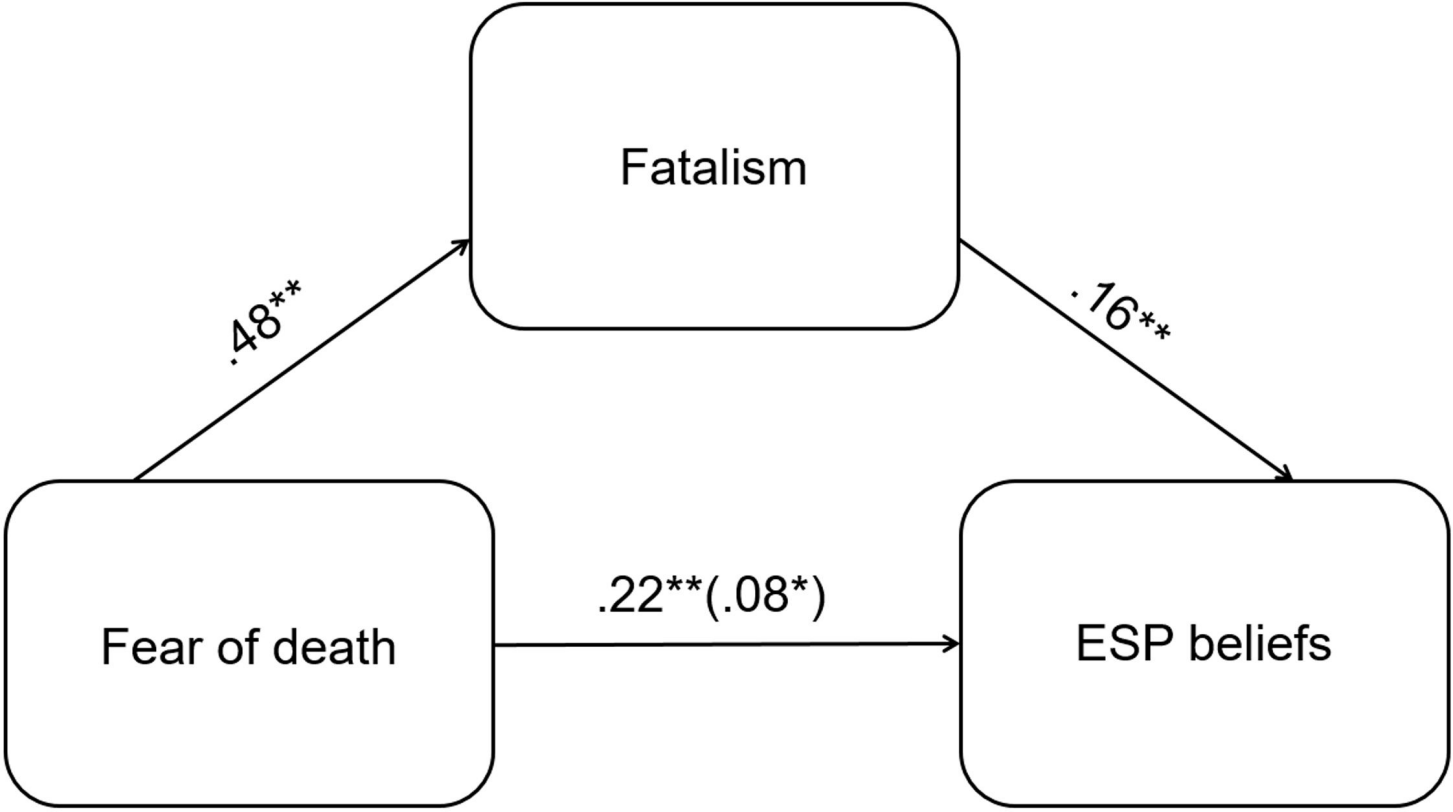
Relations between ESP beliefs, fatalism and fear of death.

We further explored the relations among the variables using PROCESS macro for SPSS (Hayes, 2013). Since only the fatalism subscale correlated with ESP beliefs, we entered this variable in the mediation analysis. Mediation analysis showed that fear of death and IPS fatalism subscale explained 12% of the variance in ESP belief scores, *F*(209, 2) = 14.07, *p* < .001, (Figure 1). Death fear had both a direct (*b* = .22, *SE* = .07, 95% CI [.07, .36]) and an indirect effect on ESP beliefs, that is, this relation was partially mediated by the tendency to attribute events to chance (*b* = .08, *SE* = .03, 95% CI [.03, .15]).

We also tested the alternative model, in which fatalism is entered as the predictor and fear of death as the mediator. This model also showed partial mediation, however, the effect sizes were slightly smaller compared to the previous model: the direct path from fatalism to ESP (*b* = .16, *SE* = .05, 95% CI [.06, .27]), and the indirect path through fear of death (*b* = .06, *SE* = .02, 95% CI [.02, .10]).

### Discussion

In this study, we addressed some important motives underlying ESP beliefs. We established a positive relation of ESP belief with death anxiety as well as the propensity to attribute events in life to chance. Moreover, fatalism partially mediated the effects of death anxiety on ESP. The findings are in line with the rare studies that investigated the relationship between fear of death and forms of paranormal belief other than religious (Tobacyk & Pirttilä-Backman, 1992; Wong, 2009). These findings need to be distinguished from those related to more traditional religious beliefs that have typically exhibited a negative relation with death anxiety (e.g. Tobacyk & Pirttilä-Backman, 1992; Norenzayan & Hansen, 2006).

The present findings speak to the conclusion that ESP beliefs are at least partly driven by some fundamental existential concerns, as facing the uncertainty of existence and death. A relevant theoretical framework to understand this relation is offered by terror management theory (TMT; TMT, Greenberg, Pyszczynski, & Solomon, 1986; Pyszczynski et al., 1997; Pyszczynski, Solomon, & Greenberg, 2015; Solomon, Greenberg, & Pyszczynski, 2004). This theory posits that simple defense mechanisms as negation or rationalization do not suffice to avert fear arising from the awareness of mortality and that people, therefore, need to lean on more elaborate symbolic defenses. Complex cultural worldviews are potent enough to offer solace and a hope of individual transcendence to people who espouse them and strive to attain self-esteem within the standards they define. Religion has a particular importance as a defensive structure, owing to its direct relation with the promise of afterlife and immortality (Vail et al., 2010). For instance, it has been shown that offering proof of literal immortality (existence of an afterlife) buffers other defensive reactions to mortality reminders (Dechesne et al., 2003).

A similar logic can be extended to the role of ESP beliefs, as a sort of belief in invisible forces that speak about a reality that is beyond our senses or reason. From terror management perspective, the allure of such beliefs can be explained by the desire for transcending the limitations of the mortal self. However, since these beliefs are only loosely related to a sense of an afterlife or supernatural agents, they could not be as effective a defense as the more traditional religious beliefs. Thus, they could be driven by similar motivation but not offer the same kind of psychological protection. Since the data at hand are correlational, the exact causal pathways are not possible to establish: one the one hand, it makes sense that more fear creates more need to believe in ESP (among other things); on the other, stronger belief could also lead to more fear, which is at least partly consistent with some previous studies including people who had unusual experiences (Kennedy & Kanthamani, 1995). Moreover, both directions could, in fact, combine and result in what Wong (2009) describes a vicious circle of anxiety creating more belief, which, in turns, does not succeed in relieving the anxiety.

As the relation between ESP beliefs and fear of death was partly mediated by fatalism, i.e. belief that chance controls one’s outcomes in life, this appears to be one of the relevant concerns addressed by these beliefs. The less one feels one can control the events in life and environment, the more alluring do ESP beliefs become as at least some kind of framework for understanding reality. The present results are thus consistent with previous research relating at paranormal belief with the need for control (Irwin, 1993, 2000; Rudski & Edwards, 2007), as well as the external locus of control (Dag, 1999; Groth-Marnat & Pegden, 1998; Tobacyk & Tobacyk, 1992). The present findings further specify this locus, as well as its relations with other basic motivations. As mentioned, the exact nature of relations between fear of death and fatalism as determinants of ESP belief needs further, preferably experimental, research.

The findings are also in line with the studies done by Fritsche and colleagues (2008), showing that a need for control can underlie fear of death. However, since the mediation we established was only partial, there appear to be other relevant aspects of death fear as a basic motivation for ESP. They could also address the need for meaning in life, in suggesting the possibility that there is a wider or a transcendent reality beyond ours. This potential source of motivation should be explored in future studies.

## General Discussion

As the two studies reveal, ESP beliefs can be conceptualized and measured as a coherent and a relatively distinct set of paranormal beliefs. These beliefs appear to be more strongly related to an intuitive cognitive style than (a lack of) rationality. In line with the dual-process models of information-processing, these beliefs are thus not irreconcilable with a rational worldview, rendering the well-educated individuals susceptible or even particularly inclined to them (Pennycook et al., 2012; Rice, 2003). The findings related to the motivational underpinnings of these beliefs suggest that these beliefs have some very basic motivational foundations and also that their psychological role can be ambivalent (Wong, 2009). Apparently, ESP beliefs could be traced back to a psychological need to account for some aspects of reality that one cannot readily understand or control and perhaps an expression of the fear of the unknown. With this initial study, we hope to contribute to a more focused and elaborate study of this specific type of paranormal belief, as a complementary approach to the study of the multidimensionality of paranormal belief in general (Irwin, 2009).

A reader of literature on ESP phenomena gets easily struck by a certain duality in research that could be traced back to whether the researchers are skeptics or believers (e.g. Irwin, 2009; Kennedy, 2005). We can concur with that ESP belief can be studied regardless of whether one thinks ESP phenomena actually exist (Irwin, 2009). However, there are two inevitably controversial issues that we would like to briefly touch upon: the costs and benefits of ESP beliefs, and, relatedly, the possibility to change them.

As regards the costs and benefits of holding ESP beliefs, one important aspect is their consequences for individual well-being. Whether or not ESP phenomena are real, people might experience consolation or a sense of meaning believing in them – along the lines of the literature supporting the utility of illusions (e.g. illusion of control, Langer, 1975; Taylor & Brown, 1988, 1994). The research on this is still scarce and without conclusive evidence. We have mentioned one study in particular that explored the effects of the (self-reported) unusual experiences (Kennedy & Kanthamani, 1995) and concluded these effects are predominantly positive – related to a heightened sense of meaning, belief in an afterlife and supernatural agents. However, these positive effects were not reported by each individual and, furthermore, 45% of participants also reported that these experiences caused fear. The current findings are consistent with those less favorable outcomes: ESP beliefs can arise from a need for more certainty and control but fail to offer it. More research is clearly needed to support (or refute) this. Another important aspect of the costs-and-benefits issue is the wider socio-political consequences. Although ESP beliefs in their own might appear more intimate and less related to the political realm, they could also acquire more societal provenance. This could happen in particular under conditions of social unrest and uncertainty, as evidenced, for instance, by a proliferation of seers and magical healers in Serbia in the turbulent decade of nineties. In such circumstances, these beliefs and resulting behaviors can easily be manipulated towards political motives and aims, for instance providing alternative (e.g. supernatural) explanations for the current social events.

The second issue is the stability or, put differently, the possibility to change ESP beliefs. This is a clearly controversial issue for the parapsychologists, which would claim there is no need to change them and that the mainstream scientists should consider changing their dogmatic skepticial views (Kennedy, 2005). Although there is a possibility that skepticism can outgrow its own benefit (Blackmore, 1992) one can certainly hold that acting on any belief that defies rational reflection or scrutiny might become problematic, both for individual and for the society. The present findings do speak to the relative stability of the ESP in that they a. are not reducible to a deficit in reasoning that can easily be unlearned, b. have some basic motivational foundations. The previous attempts to educate students into a more critical stance towards paranormal claims have had at least some short-term favorable effects in developing more skepticism (Banziger, 1983; Gray, 1985; Manza et al., 2010). Such efforts might also benefit from an open discussion of the motives and needs that these beliefs fulfill, with the expectation that achieving insight into one’s beliefs would make a person more reflective and less uncritically attached to them. This expectation should be tested experimentally.

### Limitations of the Present Study and Suggestions for Further Investigations

The present studies have important limitations. As they were correlational in design, they do not allow drawing any causal conclusions and further experimental studies are needed to more clearly establish these. Also, the present studies revealed only some of the important determinants of ESP beliefs, and future studies should include other variables. Other determinants might be related to the social context, both wider socio-political contexts (McCann & Stewin, 1984) as well as more immediate interpersonal context (Markovsky & Thye, 2001). For instance, participants were more likely to express belief in the energy of a pyramid vs. an ordinary shaped cardboard box to preserve the freshness of fruit when other people also expressed it (Markovsky & Thye, 2001).

The issue of cross-cultural specificity of paranormal belief has yet to be addressed thoroughly, although some of the initial studies do suggest a degree of cultural specificity (Tobacyk & Pirttilä-Backman, 1992; Tobacyk & Tobacyk, 1992). The present studies are also interesting in that they study ESP beliefs in a specific socio-cultural setting, one characterized by high and rising levels of religiosity (Blagojević, 2013; Dušanić, 2007). Therefore, it might be interesting to study ESP beliefs in relation to religious beliefs and identification in future studies. With these initial studies, we hope to have contributed to understanding of the psychological bases of ESP belief, as a specific and a highly prevalent form of paranormal belief in the contemporary society.
